# Stress-induced changes in group behaviour

**DOI:** 10.1038/s41598-019-53661-w

**Published:** 2019-11-20

**Authors:** Tanja K. Kleinhappel, Thomas W. Pike, Oliver H. P. Burman

**Affiliations:** School of Life Sciences, University of Lincoln, Lincoln, LN6 7DL UK

**Keywords:** Animal behaviour, Social behaviour

## Abstract

Testing animals in groups can provide valuable data for investigating behavioural stress responses. However, conventional measures typically focus on the behaviour of individual animals or on dyadic interactions. Here, we aimed to determine metrics describing the behaviour of grouping animals that can reveal differences in stress responses. Using zebrafish (*Danio rerio*) as a model, we observed replicated shoals both immediately and 24 hours after exposure to a novel environment, as an assessment of temporal change in response to an acute stressor. We quantified various standard behavioural measures in combination with metrics describing group structure, including different proximity, social, and spatial metrics. Firstly, we showed a high collinearity between most of the analysed metrics, suggesting that they describe similar aspects of the group dynamics. After metric selection, we found that under acute stress shoals had significantly higher shoal densities, a lower variation in nearest neighbour distances and were in closer proximity to the walls compared to the same groups tested 24 hours later, indicating a reduction in acute stress over time. Thus, the use of group metrics could allow for the refinement of behavioural protocols carried out in a range of research areas, by providing sensitive and rich data in a more relevant social context.

## Introduction

Animals in captivity experience stressors in a wide range of contexts, including actual physical challenges, such as changes in housing conditions, or just a threat of such a challenge, like the approach of a dominant conspecific or a human handler (reviewed in^1^). Chronic, but also acute, stressors typically result in a cascade of physiological and behavioural changes in the animal which can threaten its welfare and survival^1^. The reliable assessment of stress responses is therefore of great interest in a variety of different research fields, including, behavioural neuroscience^2^, biomedical and preclinical research^3–5^, psychopharmacology^6,7^ and welfare science^8–10^.

Studies investigating these physiological and behavioural responses to stress often rely on animals being removed from their social environment and being placed in individual testing environments (e.g.^5,11,12^). However, most of the species of interest are social animals and are therefore routinely housed with other conspecifics in order to maximise their welfare (reviewed in^13,14^), allowing them to interact and display a variety of natural social behaviours. As a consequence, separating individual subjects from their social group for experimentation (e.g.^5,11,12^) prevents them from expressing their full range of behaviours^15,16^. For example, social interactions and inter-individual proximity may give earlier indications of stress within a group than individual behaviours, as more individuals are more alert to changes in the environment (e.g. predator threats^17^). Social separation may also increase the stress of the individual under observation due to the acute effect of a group-housed animal being alone^18^, which may be helpful in some contexts (e.g. when studying anxiolytic treatments^19^), but less so in others when animals might be better studied in social groups (e.g. when studying anxiogenic treatments, or treatments affecting social behaviour). In addition, there is an increasing emphasis on the development of testing protocols that use more ‘naturalistic’ conditions. This is supported by the recent developments and applications of novel techniques that allow and promote the assessment of individuals in more natural group settings (e.g.^15,20^).

Despite animals displaying a wide range of behaviours when part of a group, when animals are tested in groups, for instance when investigating welfare indicators under different husbandry conditions, the range of behaviours recorded in studies is often restricted to those related to aggression (e.g. chasing/biting/fighting^21–23^), allogrooming and play^24–26^, or subjective scores (e.g.^6^). This often limits observations to dyadic interactions, missing out on the greater wealth of data and interactions involved within a group^27^. In research areas, such as behavioural ecology, researchers commonly use a variety of different group metrics in order to describe group responses to changes in their physical or social environment (e.g.^28–30^), and these, or variations of them, could be of advantage for assessing behavioural stress responses of groups. For instance, metrics quantifying group cohesion and compactness (e.g. inter-individual and nearest neighbour distances) are commonly used to investigate group organisation and responses to a variety of social and environmental factors^31–34^, including group structure changes due to predator cues^29^ and differences in group dynamics between genetic strains^35^. Furthermore, metrics derived from social network analysis, can be used to assess detailed patterns of social interactions within groups, such as the presence and size of subgroups or the overall sociability of animals within the testing environment (e.g. degree in which individuals are associating with each other; described in^28,36^) at specific time intervals over the observation period without the necessity of individual identification. The overall dispersal of individuals (e.g. the area surrounding all individuals^30^), in conjunction with spatial positions within the testing environment (i.e. thigmotaxis, the propensity to avoid the centre of a testing environment and stay or move in close proximity to the boundaries), could also be important indicator of stress, and the latter has been repeatably shown to predict stress in individually tested animals^5,37^.

The aim of this study was therefore to identify different metrics visible in groups of animals that could reveal differences in their temporal response to acute stress. Using zebrafish (*Danio rerio*) as a model, we observed both the immediate behavioural response of replicated shoals of fish to a novel environment (i.e. an acute stressor) and then 24 hours later, when it was predicted that responses to the acute stressor would have dissipated. Stressful situations including a novelty element (e.g. novel environment) have been shown to trigger the expression of characteristic and robust physiological and behavioural stress responses upon initial exposure^5^ in a variety of different species (e.g. mice^3^, sheep^38^, fish^39^). In fish, researchers typically analyse behaviours such as time spent on the bottom of the tank, latencies to enter the upper half or transitions into the upper half of the experimental tank and erratic movements, as they have all been linked to stress in fish^40–42^. In addition to these behavioural changes, also physiological changes, such as an increase in whole-body cortisol levels can be observed in fish being individually placed into a novel experimental tank (e.g.^41,43^). In the present study we firstly analysed whole-body cortisol levels of fish to confirm that the novel testing tank induced stress in the shoals as it has been shown for individual fish (e.g.^43^). Secondly, behavioural metrics, and non-dyadic metrics quantifying spatial positions and group structure were analysed, describing different aspects of group dynamics, in order to test whether they can reveal differences between the immediate exposure to an acute stressor and behavioural expressions 24 hours later, emphasising the additional range of informative data potentially available to researchers in various research contexts.

## Methods

### Animals and housing

Adult wildtype zebrafish (*Danio rerio*), obtained from a home aquarium supplier (Aquatics to your Door, UK), were housed in mixed-sex groups in the aquatics facility at the University of Lincoln (UK). Shoals were kept at a density of approximately 1fish L^−1^ in unenriched holding tanks measuring 52 × 44 × 31 cm and filled with 35 l of dechlorinated and UV sterilised water. Water was maintained at a constant temperature of 24 ± 1 °C. The photoperiod was 12:12 light - dark cycle (on: 6:00 h, off: 18:00 h) provided by ceiling-mounted fluorescent lights. Fish were fed daily to saturation with defrosted Chironomidae larvae (bloodworms). To maintain similar hunger levels for fish during behavioural testing they were fed one hour before the start of the observations, which always took place at 10 am for all groups.

### Apparatus and behavioural testing

To investigate behavioural expressions of stress, replicated groups, each consisting of 7 unfamiliar fish, were exposed to a novel testing tank and their immediate behavioural responses were observed as well as behaviours 24 hours later. A total of 184 fish were used which, at the start of the study, had an average standard body length of 36.98 mm (±2.24 SD). A maximum of 5 groups were tested at the same time, and each fish was tested only once. A total of 23 shoals were used as replicates in a repeated measures design.

The experimental tanks were glass aquaria measuring 45 × 25 × 25 cm (L × W × H) and filled with aerated dechlorinated water to a depth of 20 cm (22.5 litres). Experimental tanks were located in the same room as the holding tanks. The long sides of each experimental tank were covered with opaque (white) window film, to visually isolate the tanks from each other. Before the start of an experimental session, one fish was carefully netted from each of the 7 different holding tanks allocated to this study (to ensure equal unfamiliarity at the start of the experiment^44^) and placed into a beaker containing 500 ml of dechlorinated water. Fish were then gently released into the centre of the testing tank and the first data collection session started immediately. Shoals were filmed for a duration of 30 min with two cameras. A Raspberry Pi Camera Module V2 connected to a Raspberry Pi 3 Model B was positioned at the front (short side) and a GoPro Hero3 white was mounted above so it looked down onto the tank. Both cameras were remotely set to start recoding at a specified time (briefly before the fish were released into the testing tank and at the same time the following day), so that fish in the experimental tanks were not disturbed by the experimenter switching on the cameras manually. After 30 min, one fish from each shoal was carefully netted and immediately euthanised with an overdose of buffered MS-222 (E10521, Sigma-Aldrich, UK; e.g.^43^). The fish was then blotted on a paper towel to remove excess anaesthetic and then frozen at −80 °C in individual screw top test tubes for later cortisol analysis. Sampling each fish took less than 30 s, which is below the observed peak of whole-body cortisol arising between 9 and 15 min after a net handling stressor^45^, ensuring that whole-body cortisol concentrations seen in this study were related to the treatment and not the handling stress occurring before the fish was euthanised. Another unfamiliar fish from one of the housing tanks was then added to each shoal to maintain a group size of 7 fish. After 24 hours, shoals were filmed for a further 30 min as described above. At the end of the 30 min another fish from each shoal was carefully netted and euthanized for later cortisol analysis. All remaining fish were transferred back into new housing tanks (i.e. not containing experimental fish) for use in future work.

Sample size in this study was chosen using a paired t-test power analysis based on an estimated upper range of variance of whole-body cortisol data observed in adult zebrafish (e.g. 2.2–5.6 ng g^−1^ depending on the study^40,43^) with a minimum effect size of 4 ng g^−1^ (shown in^45^ between stressed and non-stressed individuals), such that the power for detecting differences in whole-body cortisol concentration between stressed and non-stressed fish is ≥90%. This study followed the ARRIVE guidelines^46^ (see Table S3 for checklist) and all methods used adhered to the ASAB Guidelines for the Use of Animals in Research and gained local institutional ethical approval by the Research Ethics Committee of the University of Lincoln (UID CoSREC211).

### Cortisol assay

As the concentration of waterborne cortisol with 7 fish in a water volume of 22.5 litres would have been below the detection limit for an ELISA or a radioimmunoassay (RIA), and isolating individual fish in smaller volumes of water after the observation period would induce confinement and isolation stress^47^, whole-body cortisol was extracted from fish using the method described in^43^. Fish were weighed (µg) and sectioned into smaller pieces to facilitate homogenisation. Samples were homogenised with 2 ml of phosphate buffered saline (PBS) and the rotor blades were washed with an additional 1 ml of PBS. Both the fish homogenate and the wash were collected in a disposable screw top test tube and 5 ml of diethyl ether were added. Samples were vortexed for 1 min and then placed in the centrifuge at 7000 g for 15 min. After centrifugation the top organic layer containing the cortisol was placed in a separate disposable screw top test tube. The extraction procedure was repeated twice more on the homogenate to ensure maximal cortisol extraction. The pooled extract from each fish was placed overnight in a fume hood to allow the ether to evaporate at room temperature. The dried extracts were stored at −80 °C. A commercially available ELISA (Enzo Life Sciences, Cortisol ELISA kit, ADI-900-071) was performed to quantify cortisol concentrations. Extracts were reconstituted in 2 ml of diethyl ether and 125 µl samples were pipetted into a test tube and used in the assay. The test tubes were placed in a fume hood to evaporate the solvent and the dried extract was dissolved in 250 µl of assay buffer, vortexed, left to sit for 5 min and vortexed another two times. Reconstituted samples were run in the assay immediately using the method described in the product manual. Samples were replicated across plates with standards being replicated on each plate. One sample had a cortisol concentration below the detection limit (0.0567 ng ml^−1^) on both plates and was assigned a cortisol concentration of 0 ng ml^−1^ for the analysis. Cortisol concentration were expressed as ng g^−1^ body weight.

### Behavioural analysis

#### Behavioural metrics

Possible behavioural expressions of stress were assessed using the 30 min video footage from the front positioned camera for both observation times (see Table S1 for definitions). Each video was cut into 10 s long video clips, resulting in a total of 180 videos for each group for each condition, each of which was analysed for the presence and absence of standard behavioural measures used for fish tested individually (e.g.^43^; Table S1). In addition, as fish were tested in groups, we were able to analyse the presence and absence of group-based behaviours described for zebrafish (see^48^; Table S1). A behaviour was counted as present if at least one fish of the shoal was performing it during a 10 s clip. For all the behavioural measures ten percent of the total number of video clips (828 videos) were analysed a second time in a random order (blind to the condition) to test for intra-observer reliability. For all behaviours the percentage of agreement was greater than 95%, with kappa statistics varying from 0.75 to 0.94 depending on the behaviour (Table S1). Behaviours that were present for less than 10% over the combined observation period of both treatments were removed from the data set for later analysis as they were not representative for the testing conditions. Furthermore, species specific behaviours (i.e. circling and display behaviour; Table S1) that are only described for zebrafish were also removed retrospectively to focus only on behaviours generally present in fish, unrelated to the species.

#### Metrics of spatial position and group structure

In addition to the behaviours, images were extracted from the videos taken from above the tank in 10 s intervals, resulting in a total of 180 frames for each group for each observation time. Individual 2D positions of all fish in the shoals were manually extracted from these frames using custom-written Matlab (MathWorks, Natick, MA) code. The camera calibrator app from the vision toolbox in Matlab was used to estimate the camera lens distortion parameters using 10 images of the calibration pattern (a black and white checkerboard) at different angles. The resulting camera parameters were then used to correct the coordinates. The corrected fish positions were used to compute different spatial metrics (see Table S2 for definitions) as well as the Euclidean distance between all individuals to analyse commonly used proximity metrics in shoaling fish (see Table S2 for definitions). Finally, binary association matrices (e.g.^28^) were constructed for each frame to compute different social metrics (see Table S2 for definitions). Two fish were assumed to be associating if they were within two body lengths of each other (i.e., twice the mean body length of all fish from the same group). This distance is within the range of inter-individual distances observed in free‐ranging shoals^49^ and has previously been used to characterise social interactions in fish^50–52^.

### Statistical analysis

All analyses were conducted in R v. 3.4.4 (R Core Development Team). A linear mixed-effects model (using the lmer function of the lme4 package^53^) was conducted to test if the cortisol concentration differed as a function of the testing condition with group as a random effect to control for repeated sampling of fish from each group. Cortisol concentration was log transformed to ensure normally distributed residuals in the model.

In order to identify metrics that describe different aspects of the shoaling dynamics within our testing population we aimed to select a subset of metrics that were uncorrelated with one another (specifically those with a correlation coefficient of |r| < 0.2, as we considered this to be a weak to negligible correlation), while retaining at least one metric from each of the analysed categories (behavioural, social, proximity, spatial metric). If two metrics were correlated, we selected one of them for inclusion in the final set. Pairwise correlations between all analysed behavioural, spatial, proximity and social metrics was assessed using Spearman correlations for non-normally distributed continuous metrics and Pearson correlations for other data type correlations^54^. A correlation matrix including all metrics was produced using the corrplot package in R^55^.

After metric selection, individual linear mixed-effects models were fitted by maximum likelihood to determine if the average shoal density, CV nearest neighbour distance and the distance of the shoal to the nearest wall differed as a function of the observation condition. CV nearest neighbour distance was log transformed to ensure normally distributed residuals in the model. In all models group was added as a random effect to control for repeated observations of the groups in both conditions. Individual generalized mixed-effects models (using the glmer function of the lme4 package) were fitted by maximum likelihood (Laplace Approximation) and a binomial family to determine if the proportion of erratic movement and individuals being in the top half of the testing tank differed as a function of the observation conditions, with the behaviours being present or absent as the binomial response variable. Group identity was added as a random effect to control for the repeated observation of groups in both conditions.

Significance in all models was assessed by comparing the full model to null models lacking the fixed effect of interest using likelihood ratio tests (Crawley, 2011). All p-values were adjusted for multiple testing using Bonferroni correction.

## Results

Immediately after exposure to novelty individuals exhibited on average significantly higher whole-body cortisol concentrations (mean ± SE: 40.2 ± 6.0 ng g^−1^) compared to individuals observed 24 hours later (mean ± SE: 19.9 ± 4.3 ng g^−1^; χ^2^(1) = 24.85, p < 0.001).

**Figure 1:**
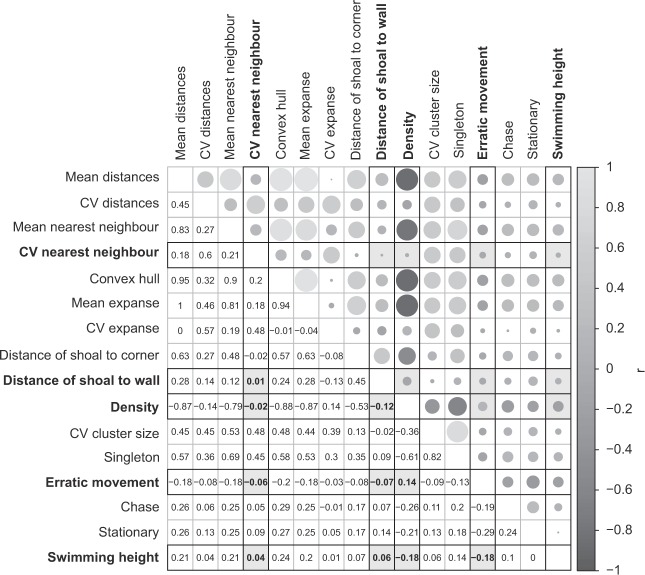
Correlation matrix between all the analysed metrics in this study, with non-correlated metrics and their correlation coefficients highlighted in bold and grey background shading. The areas of the circles correspond to the value of the correlation coefficient (r), with smaller circles representing smaller r values. The legend on the right of the correlogram shows the correlation coefficients with their corresponding colours.

**Figure 2:**
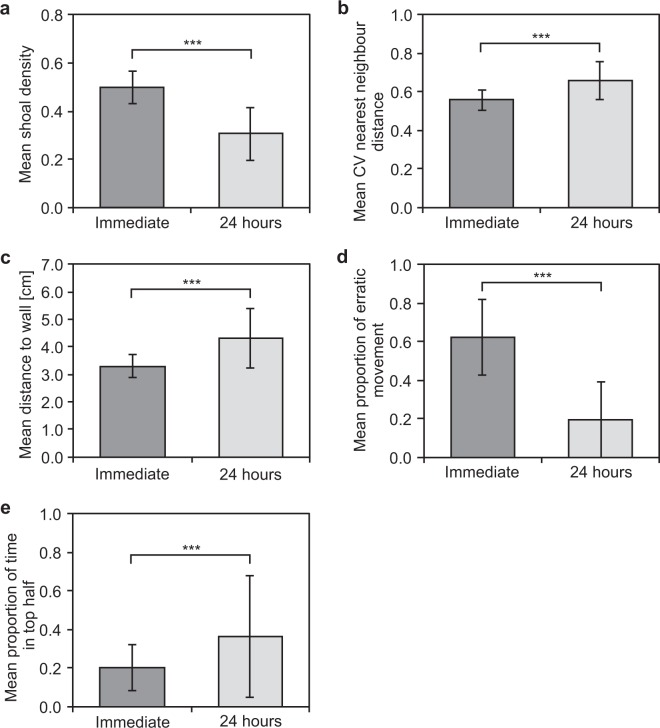
Mean ± SD of **(a)** shoal density, **(b)** CV nearest neighbour distance, **(c)** distance to the nearest wall, and the proportion of time at least one fish in the shoal **(d)** exhibits erratic movement, and **(e)** is in the top half of the testing tank in the immediate and 24 hours condition. Asterisks (*) denote significant differences between groups: ***p < 0.001.

The correlation matrix indicated the presence of high collinearity between the analysed metrics (Fig. 1) and only five non-correlated metrics remained after the selection process (see Table 1 for description; Fig. 1). Analysed metrics revealed that immediately after exposure, shoals had a significantly higher average shoal density (χ²(1) = 2001.9, p < 0.001; Fig. 2a), a smaller average CV in nearest neighbour distance (χ²(1) = 165.8, p < 0.001; Fig. 2b) and a shorter average distance to the nearest wall (χ²(1) = 370.3, p < 0.001; Fig. 2c) compared to the same shoals observed 24 hours later. Furthermore, fish exhibited a higher average proportion of erratic movement (χ²(1) = 1763.9, p < 0.001; Fig. 2d) and spent less time in the top half of the testing tank (χ²(1) = 320.9, p < 0.001; Fig. 2e) under acute stress compared to 24 hours later.

**Table 1:** Name and description of the non-correlated metrics used in this study.

Metric	Description
Distance of shoal to wall	A spatial metric, defined as the distance of the centre of mass of the shoal to the nearest wall. Measures a shoals’ tendency to be close to the walls in the testing tank (i.e. thigmotaxis).
CV nearest neighbour	A proximity metric, defined as the coefficient of variation of the nearest neighbour distance of all individuals within the group. Measures the variation in within shoal structure.
Density	A social metric, defined as the number of individuals associating (i.e. individuals within 2 body length) divided by the total number of possible associations within the shoal. Measures the sociability of the groups.
Erratic movement	A behavioural metric, defined as the presence of at least one fish in the shoal displaying sharp changes in direction or velocity; repeated rapid darting/dashing
Swimming height	A behavioural metric, defined as the presence of at least one fish of the shoal being in the top half of the testing tank

## Discussion

Metrics describing spatial position and group structure in combination with behavioural measures were significantly different immediately after exposure to the novel environment (i.e. acute stressor) when compared to 24 hours later. Exposure to novelty is commonly used to study acute stress responses in animals^4,5,56^, although in a majority of studies animals are tested individually^3,40,42^. Firstly, the results of this study showed that on average whole-body cortisol concentration was significantly higher immediately after groups were exposed to the novel tank. In addition, standard behavioural measures used for individually tested fish, including animals displaying erratic movements or being observed at the bottom half of the testing tank, significantly differed between the two conditions, with both behaviours being present more frequently immediately after exposure to the novel tank. In combination, these results demonstrate that novelty (in this case a novel testing tank) can be an acute stressor for individuals tested in shoals, as has been shown when individuals are tested alone (e.g.^41,43^). In relation to this, the lower whole-body cortisol concentration, and the reduced presence of erratic movement as well as the increased presence of fish in the top half of the testing tank indicate lower acute stress levels in groups 24 hours after initial exposure to novelty. Our results are supported by studies looking at stress responses of fish in the context of alarm pheromones or anxiogenic treatments, showing that control fish exhibit fewer erratic movements and spend more time in the upper half of the testing tank compared to individuals experiencing the test treatments^41,43^. Finally, and more interestingly, measures describing group structure and spatial position of shoals of fish were also significantly different between the two conditions, suggesting that group metrics can provide sensitive measures for assessing stress responses.

The high collinearity between most of the analysed metrics (Fig. 1) suggested that they capture similar aspects of the group dynamic and structure. Indeed, proximity measures such as convex hull (the smallest possible area containing all fish within the shoal^30^), expanse (the average distance of all fish to the centre of mass of the shoal^32^) and nearest neighbour distance (distance between all fish and their nearest individual^33^) all measure similar aspects of group cohesion and compactness and are therefore not likely vital to assess within the same study. These measures were also highly correlated with the social metrics assessed in this study, such as the presence of subgroups and shoaling density (Fig. 1). Social metrics in fish are commonly computed using association distances (i.e. proximity^50,52,57^), and are therefore very similar to proximity measures in this study, however this might not be correlated in other animal species when computed using behaviours such as aggression as interaction measure rather than proximity (e.g.^58^).

Zebrafish are a highly social species which readily exhibit shoaling behaviour^59^. Consequently, it is not unexpected that metrics describing group structure and individual associations can be affected by acute stress. Indeed, shoals in our study exhibited higher shoaling densities and a smaller variation in their nearest neighbour distances immediately after exposure to the novel tank, emphasising the importance of assessing within-shoal structure when measuring stress responses in groups. These findings are comparable to other studies showing that (negative) stressors, such as the presence of predators or alarm substances can increase shoal cohesion^29,60,61^. Furthermore, differences in shoaling configurations (i.e. shoaling vs. schooling) depending on the familiarity to a testing tank have also been shown in zebrafish, with shoals spending less time schooling (and more time shoaling) over time^34^. The larger variation in nearest neighbour distance 24 hours after exposure to the acute stressor found in our study is comparable to this finding as it indicates less cohesive (and possibly less polarised) group structures.

Also, spatial positions of fish shoals within the experimental tank significantly differed between the conditions. Thigmotaxis, the tendency of animals to avoid the centre of a testing environment, has numerous times been shown as an anxiety and stress response of animals^5,37^. Also, in this study, the average distance of the centre of the shoal to the nearest wall was smaller in groups under acute stress compared to 24 hours later. Finally, as mentioned before, general activity levels of individuals within the shoals, i.e. the presence or absence of erratic movement, and swimming height of individuals, also significantly differed depending on the testing condition, showing that fish displayed more erratic movement and are more often observed at the bottom of the testing tank when experiencing acute stress. This is in line with studies on individually tested fish that found that animals exhibit an increase in activity levels during stressful situations which then decrease after recovering or habituation^5,41^. Similarly, Bains *et al*.^15^ showed an increase in mouse activity, with their automated home cage analysis system, depending on external, possibly stressful, events.

It is important to mention that groups in this study were tested under negatively valenced conditions^62^. Yet, similar behavioural changes in group dynamics might also occur in response to positively valenced conditions, for instance during, or when individuals anticipate, feeding. Furthermore, the impact of the current housing conditions, for example being enriched or un-enriched, as was the case for our population of fish, could also impact on shoal dynamics in different testing conditions, as, for instance, enriched housing conditions have been shown to enhance recovery from stressful stimuli in fish^63^. Both areas deserve further attention.

Overall our study highlights the importance of taking social group dynamics into account when studying stress in animals, which can be relevant in a diverse range of areas including neuroscience, psychopharmacology, behavioural and cognitive sciences. Social interactions are central to group living animals and, as such, should be central when studying stress responses expressed by animals in different contexts. Social interactions have been shown to be the results of genetic, epigenetic, endocrine, and neural mechanisms^64^, and therefore can be valuable targets for various pharmacological compounds of interest. For example, anxiolytic and anxiogenic substances could be effective in addressing anxiety when tested in isolated individuals but be found to additionally disrupt social organisation in animal groups when tested in a social context. Metrics describing group structure and spatial positions could also substantially impact on the selection of indicators for assessing welfare in group-housed animals in different stressful and non-stressful situations, or be used in addition to standard testing protocols of sociability^65^. They can add valuable information for studies investigating different biological aspects of how stress can affect social behaviours, emotions and cognition, and have the potential of being used for the (early) detection of positive and negative changes in group dynamics in various research contexts. In line with this, we have shown that groups can exhibit behavioural stress responses that are only emergent from social interactions and associations, highlighting that behaviours based on the highly responsive structure of animal groups could potentially allow us to distinguish different aspects and underlying mechanisms in a more relevant and translatable social context.

## Supplementary information

Supplementary material

R script

Dataset

## Data Availability

All data generated during this study is included as supplementary data file.
