# Supplementary material for: Stress-induced changes in group behaviour
Source: Sci Rep. 2019 Nov 20;9:17200. doi: 10.1038/s41598-019-53661-w (PMC6868208; doi:10.1038/s41598-019-53661-w)
Supplement: Supplementary file 1 — Supplementary material [file 41598_2019_53661_MOESM1_ESM.pdf]

Stress-induced changes in group behaviour - Supplementary material

Tanja K. Kleinhappel, Thomas W. Pike & Oliver H. P. Burman

Joseph Banks Laboratories, School of Life Sciences, University of Lincoln, Lincoln, LN6 7DL, UK

Supplementary Table 1. Definition of behavioural metrics analysed in this study (Kalueff et al., 2013) with intra-observer reliability, showing the percentage of agreement Cohen's kappa, z statistic and p-value.

**Behaviour**	**Description**	**% agreement**	**Cohen's kappa**	**z-statistic**	**p-value**
Stationary	At least one fish in the shoal is not moving for at least 1 second (can include freezing, resting, etc.)	97.1	0.941	27.1	<0.0001
Erratic movements	At least one fish in the shoal exhibits sharp changes in direction or velocity; repeated rapid darting/dashing	96.6	0.931	26.8	<0.0001
Dart	At least one fish in the shoal exhibits a single fast acceleration in one direction	97.0	0.78	22.5	<0.0001
Chase	At least one fish in the shoal is continuously charging (see definition below) while the opponent flees (following another fish)	97.8	0.908	26.1	<0.0001
Charge	At least one fish in the shoal is moving towards a second fish increasing acceleration, while second fish avoids the first (the first fish does not follow)	99.4	0.862	24.8	<0.0001
Attack	At least one fish in the shoal exhibits a short bout of swimming directed at an opponent (with or without physical contact)	97.8	0.836	24.1	<0.0001
Circling	At least one pair of fish is showing a lateral display behaviour circling each other (can be part of aggression as well as courtship)	99.3	0.766	223	<0.0001
Display	At least one fish is raising its dorsal fins, extends its caudal fins, and darkens in colour in response to other fish in the shoal (as a result of aggression or courtship behaviour)	97.5	0.875	25.2	<0.0001
Swimming height	At least one fish in the shoal is in the bottom half of the testing tank	97.5	0.932	26.8	<0.0001

Supplementary Table 2. Definitions and description of the different spatial, proximity and social metrics.

	**Metric**	**Definition**	**Description**
**Spatial metrics**	Distance of shoal to corner	Distance of the centre of mass of the shoal to the nearest corner	Measures a shoals' tendency to be in a corner of the testing tank (i.e. thigmotaxis)
	Distance of shoal to wall	Distance of the centre of mass of the shoal to the nearest tank wall	Measures a shoals' tendency to be close to the walls in the testing tank (i.e. thigmotaxis)
**Proximity metrics**	Convex hull	The smallest possible area containing all fish within the shoal (e.g. Stienessen and Parrish, 2013)	Measures the overall distribution of individuals within the testing tank
	Mean expanse	The average distance of all fish to the centre of mass of the shoal (e.g. Huth and Wissel, 1992)	Measures the compactness and cohesion of the group
	CV expanse	The coefficient of variation of the expanse	Measures the variation of individuals being close to the shoal centre
	Mean inter-individual distances	The average distance between all individuals within the shoal (e.g. Miller and Gerlai, 2012)	Gives information about the within shoal structure of the group
	CV inter-individual distances	The coefficient of variation of all distances between all individuals within the shoal	Measures the variation of the proximity of individuals, e.g. high cv indicates that fish are not evenly spaced between each other
	Mean nearest neighbour distance	The average of the distance between all fish and their nearest individual (e.g. Miller and Gerlai, 2012)	Gives information of the within shoal structure, as some individuals can be far apart but still close to at least one other individual
	CV nearest neighbour distance	The coefficient of variation of the nearest neighbour distance of all individuals within the group	Measures the variation in within shoal structure.
**Social metrics**	Density	The number of individuals associating (i.e. individuals within 2 body length) divided by the total number of possible associations within the shoal	Identifies the proportion of fish in association, a measure of sociability (i.e., the tendency to interact with other fish)
	CV cluster size	The coefficient of variation of the number of individuals within a cluster, calculated using the *igraph* package and the component function in R 3.4.3.	Identifies the variation of the size of subgroups within the shoals
	Singleton	A cluster containing a single individual	Identifies single individuals that are separated from all other individuals within a group

Supplementary Table 3. ARRIVE Guidelines (Kilkenny et al., 2010) – checklist

	Item	Recommendation		Additional comments
Title	1	Provide as accurate and concise a description of the content of the article as possible.	✓	
Abstract	2	Provide an accurate summary of the background, research objectives, including details of the species or strain of animal used, key methods, principal findings and conclusions of the study.	✓	
**Introduction**				
Background	3	a. Include sufficient scientific background (including relevant references to previous work) to understand the motivation and context for the study, and explain the experimental approach and rationale.<br>b. Explain how and why the animal species and model being used can address the scientific objectives and, where appropriate, the study's relevance to human biology.	✓	
Objectives	4	Clearly describe the primary and any secondary objectives of the study, or specific hypotheses being tested.	✓	
**Methods**				
Ethical statement	5	Indicate the nature of the ethical review permissions, relevant licences (e.g. Animal [Scientific Procedures]	✓	The study followed the ASAB Guidelines for the Use of Animals in Research and gained

		Act 1986), and national or institutional guidelines for the care and use of animals, that cover the research.		local institutional ethical approval (UID CoSREC211)
Study design	6	For each experiment, give brief details of the study design including:<br>a. The number of experimental and control groups.<br>b. Any steps taken to minimise the effects of subjective bias when allocating animals to treatment (e.g. randomisation procedure) and when assessing results (e.g. if done, describe who was blinded and when).<br>c. The experimental unit (e.g. a single animal, group or cage of animals).<br>A time-line diagram or flow chart can be useful to illustrate how complex study<br>designs were carried out.	✓	Details of all relevant points are given in the manuscript, including within treatment conditions, random individual allocation to testing groups, intra-observer reliability, and the use of groups as the experimental unit.
Experimental procedures	7	For each experiment and each experimental group, including controls, provide precise details of all procedures carried out. For example:<br>a. How (e.g. drug formulation and dose, site and route of administration, anaesthesia and analgesia used [including monitoring], surgical procedure, method of euthanasia). Provide details of any specialist equipment used, including supplier(s).<br>b. When (e.g. time of day).<br>c. Where (e.g. home cage, laboratory, water maze).<br>d. Why (e.g. rationale for choice of specific anaesthetic, route of administration, drug dose used).	✓	Details of experimental procedures are given in the manuscript, including the method of whole-body cortisol analysis, time and location of testing, and rationale of choosing a novel tank treatment for inducing mild stress.

Experimental animals	8	a. Provide details of the animals used, including species, strain, sex, developmental stage (e.g. mean or median age plus age range) and weight (e.g. mean or median weight plus weight range).<br>b. Provide further relevant information such as the source of animals, international strain nomenclature, genetic modification status (e.g. knock-out or transgenic), genotype, health/immune status, drug or test naïve, previous procedures, etc.	✓	All available details of the zebrafish used in this study are included in the manuscript (e.g. aquarium supplier, mixed-sex groups of adults, standard lengths at time of testing)
Housing and husbandry	9	Provide details of:<br>a. Housing (type of facility e.g. specific pathogen free [SPF]; type of cage or housing; bedding material; number of cage companions; tank shape and material etc. for fish).<br>b. Husbandry conditions (e.g. breeding programme, light/dark cycle, temperature, quality of water etc for fish, type of food, access to food and water, environmental enrichment).<br>c. Welfare-related assessments and interventions that were carried out prior to, during, or after the experiment.	✓	All details of housing and husbandry are provided in the manuscript (e.g. stocking density, feeding regime, temperature, light cycle, etc.)
Sample size	10	a. Specify the total number of animals used in each experiment, and the number of animals in each experimental group.<br>b. Explain how the number of animals was arrived at. Provide details of any sample size calculation used.	✓	Details are given in the manuscript, including the total number of animals used, the number of groups, the number of individuals used for whole body cortisol analysis.<br>Sample size was derived by using a paired sample t-test power analysis with estimated variance and effect size derived from published

		c. Indicate the number of independent replications of each experiment, if relevant.		data assessing whole-body cortisol concentrations in adult zebrafish.
Allocating animals to experimental groups	11	a. Give full details of how animals were allocated to experimental groups, including randomisation or matching if done.<br>b. Describe the order in which the animals in the different experimental groups were treated and assessed.	✓	Details about how animals were allocated into the different groups is given in the manuscript.
Experimental outcomes	12	Clearly define the primary and secondary experimental outcomes assessed (e.g. cell death, molecular markers, behavioural changes).	✓	Experimental outcomes analysed in the study are clearly defined in the manuscript.
Statistical methods	13	a. Provide details of the statistical methods used for each analysis.<br>b. Specify the unit of analysis for each dataset (e.g. single animal, group of animals, single neuron).<br>c. Describe any methods used to assess whether the data met the assumptions of the statistical approach.	✓	All statistical analysis is described in detail in the manuscript in the statistical analysis section.<br>All data was analysed using group as the unit of analysis, also models included group as a random effect in order to control for the repeated measures design.<br>Model diagnostics were applied for all models in this study in order to ensure that they were appropriate.
**Results**				
Baseline data	14	For each experimental group, report relevant characteristics and health status of animals (e.g. weight, microbiological status, and drug or test naïve) prior to treatment or testing (this information can often be tabulated).		Not applicable for this study.

Numbers analysed	15	a. Report the number of animals in each group included in each analysis. Report absolute numbers (e.g. 10/20, not 50%2).<br>b. If any animals or data were not included in the analysis, explain why.	✓	All numbers are stated in the methods.
Outcomes and estimation	16	Report the results for each analysis carried out, with a measure of precision (e.g. standard error or confidence interval).	✓	All results and graphs are presented with confidence intervals. Results of the generalised linear mixed effects model are also given with effect size estimation for each predictor variable.
Adverse events	17	a. Give details of all important adverse events in each experimental group.<br>b. Describe any modifications to the experimental protocols made to reduce adverse events.	✓	The objective of the study was to test behavioural stress responses in groups of zebrafish and therefore individuals were introduced into a novel environment that was mildly stressful for them (details given in the manuscript).<br>Nevertheless, care was taken to induce as little adverse events beside the treatment conditions. Catching individual fish for the study and after finishing the observations took never longer than 30sec and fish were never out of the water for longer than 5 sec.
**Discussion**				
Interpretation/scientific implications	18	a. Interpret the results, taking into account the study objectives and hypotheses, current theory and other relevant studies in the literature.	✓	

		b. Comment on the study limitations including any potential sources of bias, any limitations of the animal model, and the imprecision associated with the results.<br><br>c. Describe any implications of your experimental methods or findings for the replacement, refinement or reduction (the 3Rs) of the use of animals in research.		
Generalisability/ translation	19	Comment on whether, and how, the findings of this study are likely to translate to other species or systems, including any relevance to human biology.	✓	Only general behavioural metrics seen in fish were used in this study and are therefore transferrable to other fish species beside zebrafish.
Funding	20	List all funding sources (including grant number) and the role of the funder(s)<br><br>in the study.	✓	NC3Rs (NC/P001289/1)

References

Huth, A., and Wissel, C. (1992). The simulation of the movement of fish schools. *Journal of theoretical biology* 156**,** 365-385. https://doi.org/10.1016/S0022-5193(05)80681-2.

Kalueff, A.V., Gebhardt, M., Stewart, A.M., Cachat, J.M., Brimmer, M., Chawla, J.S., et al. (2013). Towards a comprehensive catalog of zebrafish behavior 1.0 and beyond. *Zebrafish* 10**,** 70-86. https://doi.org/10.1089/zeb.2012.0861.

Kilkenny, C., Browne, W.J., Cuthill, I.C., Emerson, M., and Altman, D.G. (2010). Improving bioscience research reporting: the ARRIVE guidelines for reporting animal research. *PLoS biology* 8**,** e1000412. https://doi.org/10.1371/journal.pbio.1000412.

Miller, N., and Gerlai, R. (2012). "Automated tracking of zebrafish shoals and the analysis of shoaling behavior," in Zebrafish protocols for neurobehavioral research. Springer, 217-230.

Stienessen, S.C., and Parrish, J.K. (2013). The effect of disparate information on individual fish movements and emergent group behavior. *Behavioral Ecology* 24**,** 1150-1160. https://doi.org/10.1093/beheco/art042.
