# Supplementary material for: Stress-induced changes in group behaviour
Source: Sci Rep. 2019 Nov 20;9:17200. doi: 10.1038/s41598-019-53661-w (PMC6868208; doi:10.1038/s41598-019-53661-w)
Supplement: Supplementary file 2 — R script [file 41598_2019_53661_MOESM2_ESM.pdf]

Stress-induced changes in group behaviour- R script for statistical analysis

Tanja K. Kleinhappel, Thomas W. Pike & Oliver H. P. Burman

Joseph Banks Laboratories, School of Life Sciences, University of Lincoln, Lincoln, LN6 7DL, UK

```
##load required libraries
library(lme4)
library(Matrix)

## load data file, show first lines of data
data <- read.csv('data.csv', header=TRUE)
head(data)

###linear mixed effects models

##shoaling density
m <- lmer(density ~ as.factor(condition) + (1|group), data = data, REML = F)
#likelihood ratio test: compare full model to null model lacking the fixed effect of interest
m0 <- lmer(density ~ 1 + (1|group), data = data, REML = F)
aov <- anova(m,m0)

##CV nearest neighbour distance
m <- lmer(log(cvNearNeighbour) ~ as.factor(condition) + (1|group), data = data, REML = F)
#likelihood ratio test: compare full model to null model lacking the fixed effect of interest
m0 <- lmer(log(cvNearNeighbour) ~ 1 + (1|group), data = data, REML = F)
aov <- anova(m,m0)

##shoal distance to nearest wall
m <- lmer(wallDistance ~ as.factor(condition) + (1|group), data = data, REML = F)
#likelihood ratio test: compare full model to null model lacking the fixed effect of interest
m0 <- lmer(wallDistance ~ 1 + (1|group), data = data, REML = F)
aov <- anova(m,m0)

### generalised linear mixed-effects models

##erratic movement
m <- glmer(erraticMovement ~ as.factor(condition) + (1|group), data = data, family = binomial)
#likelihood ratio test: compare full model to null model lacking the fixed effect of interest
m0 <- glmer(erraticMovement ~ 1 + (1|group), data = data, family = binomial)
aov <- anova(m,m0)

##swimming height
m <- glmer(swimmingHeight ~ as.factor(condition) + (1|group), data = data, family = binomial)
#likelihood ratio test: compare full model to null model lacking the fixed effect of interest
m0 <- glmer(swimmingHeight ~ 1 + (1|group), data = data, family = binomial)
aov <- anova(m,m0)
```
